# The successful containment of a hospital outbreak caused by NDM-1-producing *Klebsiella pneumoniae* ST307 using active surveillance

**DOI:** 10.1371/journal.pone.0209609

**Published:** 2019-02-13

**Authors:** Paola Bocanegra-Ibarias, Elvira Garza-González, Magaly Padilla-Orozco, Soraya Mendoza-Olazarán, Eduardo Pérez-Alba, Samantha Flores-Treviño, Ulises Garza-Ramos, Jesus Silva-Sánchez, Adrián Camacho-Ortiz

**Affiliations:** 1 Hospital Universitario Dr. José Eleuterio González, Universidad Autónoma de Nuevo León, Monterrey, Nuevo León, Mexico; 2 Laboratorio de Resistencia Bacteriana; Centro de Investigación Sobre Enfermedades Infecciosas, Instituto Nacional de Salud Pública, Cuernavaca, Morelos, Mexico; University of Campania, ITALY

## Abstract

The worldwide dissemination of high-risk carbapenemase-producing *Klebsiella pneumoniae* clones has become a major threat to healthcare facilities. This study describes the successful containment of a hospital outbreak caused by NDM-1-producing *K*. *pneumoniae* Sequence Type (ST) 307 using active surveillance. The outbreak began when a patient was transferred from a local hospital. After 48 hours in our hospital, a tracheal aspirate was positive for a meropenem resistant and carbapenemase-producing *K*. *pneumoniae*. All patients in the medical intensive care unit (ICU) and the neurology wards were subject to contact precautions. The hospital surfaces and devices, healthcare workers, and patients from these wards were screened by cultures. Fecal swabs were placed into broth and PCR for *bla*_KPC_, *bla*_OXA-48_, *bla*_IMP_, *bla*_VIM_, and *bla*_NDM,_ which were performed directly from the broth after 12 hours. PCRs were also performed on DNA extracted from carbapenemase-producing species from subcultured broths. Five and nine days later, two more patients’ rectal swabs tested positive. Molecular assays identified *K*. *pneumoniae bla*_NDM-1_ onto a 130-kb conjugative plasmid (IncY, IncFIIs, and IncFIIY), ST307. After the three patients were discharged, monitoring continued, and after three weeks with negative results, rectal swabbing ended. In conclusion, it was possible to contain a hospital outbreak caused by NDM-1-producing *K*. *pneumoniae* ST307 through epidemiological and microbiological surveillance. With the methodology used, the detection of NDM-type genes in fecal samples was obtained in approximately 15 hours after obtaining the fecal sample.

## Introduction

Carbapenem-resistant Enterobacteria (CRE) have been rapidly spreading worldwide and are now a significant threat in hospitals because of their high associated mortality and few antibiotic treatment options [1]. This increase in CRE is mainly due to the appearance and dissemination of carbapenemase enzymes, a specific group of β-lactamases able to hydrolyze carbapenems, which makes the strains resistant, or with reduced susceptibility, to carbapenems [2].

A high diversity of carbapenemases has been reported in *Enterobacteriaceae*, including the Ambler class A *bla*_KPC-type_, class B metallo-β-lactamases *bla*_VIM-type_, *bla*_IMP-type_, and *bla*_NDM-type_, and class D carbapenemase *bla*_OXA-48-type_ [3]. These enzymes have been identified in multiple Enterobacteria species isolated in hospitals from various regions of the world [4–6].

NDM-1-encoding plasmids were initially observed in *Klebsiella pneumoniae* and *Escherichia coli* [3], but it has been demonstrated that these plasmids can be transferred to other species, which facilitate the spread of the *bla*_NDM-1_ gene [3, 5–9], with nearly worldwide dissemination [5, 6].

The *bla*_NDM-1_ gene is located most frequently on plasmids that harbor genes conferring resistance to almost all antibiotics used to treat enterobacterial infections [3, 10]. In Mexico, *bla*_NDM-1_ has been identified in *Providencia rettgeri* [7] and *K*. *pneumoniae* [8]. Furthermore, an outbreak associated with *K*. *pneumoniae*, *E*. *coli*, and *Enterobacter cloacae* with demonstrated clonal dissemination and horizontal transfer of a 101 kb IncFII plasmid carrying the *bla*_NDM-1_ gene has been reported [9].

The worldwide dissemination of high-risk carbapenemase-producing *K*. *pneumoniae* clones has become a major threat for healthcare facilities, with sequence type (ST) 258 as the major cause of carbapenem-resistant *K*. *pneumoniae bla*_KPC-2_ and *bla*_KPC-3_ infections [11]. At present, 3,438 STs have been described (https://bigsdb.pasteur.fr/cgi-bin/bigsdb/bigscurate.pl?db=pubmlst_klebsiella_seqdef), with 72% identifying clonal groups (CG) corresponding to CG258, CG14/15, CG17/20, CG43, and CG147, with ST258 (CG258) being one of the most successful high-risk clones [11, 12]. However, the ST307 (CG307) was reported in 2008 and also emerged internationally with a higher mortality than the other clones [12].

This study describes the successful containment of a hospital outbreak caused by NDM-1-producing *K*. *pneumoniae* ST307 using active surveillance.

## Material and methods

### Hospital setting

The outbreak occurred at the Hospital Universitario Dr. José Eleuterio González, a 500-bed teaching hospital in Nuevo León, Mexico. This hospital has two adult intensive care units (ICU) units (medical and surgical with 10 beds each), one pediatric ICU with eight beds, and a neonatal ICU with 15 beds. It has approximately 23,000 admissions per year and around 200,000 emergency room visits yearly. Furthermore, this hospital receives referrals from nearby and neighboring state hospitals.

The hospital performs active surveillance of pathogens of epidemiological concern, including carbapenem-resistant *Enterobacteriaceae*. Cultures from potential sites of infection or colonization (rectal swabs) are performed within 24 h of hospital stay.

### Recognition of the outbreak

A 65-year-old patient suffered an acute ischemic stroke and was attended to in a local area hospital (P1). Upon arrival, the patient presented a deteriorated neurological status and was intubated and admitted to the ICU. The patient had not been hospitalized in the last 12 months. The family confirmed that he had not traveled, that he had not taken antibiotics for any cause, or that he had not undergone any invasive medical procedures.

After 6 days in the ICU, the patient was transferred to this institution for further treatment. On September 12, 2017, he arrived at the medical ICU and spent 24 hours in the neurology ward.

Cultures from potential sites of infection or colonization were sent after 3 days of hospital stay instead of during the first 24 h as is dictated by the standard protocol.

Aspiration was suspected, and the lower respiratory specimen and a central line were drawn.

He was administered clindamycin and ceftriaxone empirical treatments. After 48 hours, the respiratory culture (bronchoalveolar lavage) was positive for *K*. *pneumoniae* with resistance for third and fourth generation cephalosporins and meropenem. The isolate was positive for carbapenemase production.

With these results, an alert was submitted, and all patients in the medical ICU and the neurology ward were placed under standard contact precautions, including hand hygiene, universal gloving and the use of gowns. Furthermore, enhanced precautions were placed, including that all patients in the ICU, in the neurology ward and patients that had been in the same period and discharged from the ICU to other wards were put on contact precautions. The infection control unit´s staff spent additional time ensuring that these measures were implemented correctly.

### Follow-up

On September 19, 2017, 5 days after the source patient was admitted to the ICU, a second ICU patient (P2) tested positive for carbapenem-resistant *K*. *pneumoniae* from a rectal swab; the patient had severe heart failure and had no clinical signs or laboratory findings suggesting an infection. The patient succumbed due to unrelated causes 24 hours after the rectal swab was performed. The patient had a history of a congenital heart defect and did not receive any specific treatment for carbapenem-resistant bacteria.

A third patient (P3) tested positive on a rectal swab sample on October 3, 2017 –the 19^th^ day after the source patient was transferred. This patient had a history of diabetes and chronic kidney failure and was later discharged without any evidence of infection.

Patients 1, 2 and 3 shared the ICU for 6 days; they were in individual beds but had overlapping nurses, physicians, and respiratory technicians, they also shared thermometers and stethoscopes before isolation precautions were ordered.

### Monitoring and laboratory assays

Instead of routine active surveillance, that includes cultures from patients within 24 h of hospital stay; during the outbreak, the microbiological screening included culture of hospital surfaces, devices, and personnel; including patient beds (mattresses, sheets, handrails, stretchers, service and patient support tables, and rails), the areas surrounding patients (fan boards, curtains, patient pneumatic mattresses, bureaus, and doors), and healthcare workers (rectal swabs).

Additionally, rectal swabs were collected from all patients in the ICU and the neurology ward on the first and sixth days and weekly. The swabbing procedure was performed according to CDC recommendations (https://www.cdc.gov/foodsafety/outbreaks/investigating-outbreaks/specimen-collection.html).

During the ICU stay, P1 underwent bronchoalveolar lavage and was treated with tigecycline and meropenem. He was weaned from ventilator support, discharged from the ICU, and transferred to the neurology ward (second time in that ward). After the source patient´s bronchoscopy procedure, a total of 14 patients were submitted to bronchoscopy with the same bronchoscope (following standard disinfection protocols after each procedure). Samples were obtained for culture from the bronchoscopy (10 mL after cleaning of high-level disinfection and drying of the bronchoscopy) and tracheal aspirates from all patients involved.

### Laboratory assays

#### Detection of carbapenemase activity and carbapenemase-encoding genes

To process the fecal swabs, the specimens were placed into Brucella broth (1 mL) containing meropenem (2 μg/mL). Broths were cultured for 8–12 hours at 35–37°C, and after incubation, DNA was extracted directly from the broth by thermal lysis, and PCR was conducted for the KPC, OXA-48, IMP, NDM, and VIM carbapenemase-encoding genes S1 Table [13–16].

Environmental samples and the broths were subcultured onto blood and EMB agar and incubated at 35–37°C for 18–24 hours. All of the colonies with different morphologies were identified using the Bruker Biotyper mass spectrometer (Bruker Daltonics, Bremen, Germany). All of the detected *Enterobacteriaceae* were screened for carbapenemases by the CarbaNP test according to the Clinical and Laboratory Standards Institute (CLSI) guidelines [17]. For all CarbaNP positive isolates, PCR was conducted for carbapenemase-encoding genes. The positive results in PCR were confirmed by sequencing [18].

In the initial screening PCR was conducted for the KPC, OXA-48, IMP, NDM, and VIM carbapenemase-encoding genes and after we detected which carbapenemase we were dealing with, we performed only the PCR for the positive gene in subsequent fecal swabs.

#### Assays for carbapenem-resistant and carbapenemase-producing isolates

For recovered carbapenem-resistant *K*. *pneumoniae* and carbapenemase-producing isolates (from P1, P2, and P3), drug susceptibility was determined by the broth microdilution method according to the CLSI [19]. The guidelines of the European Committee on Antimicrobial Susceptibility Testing (EUCAST) version 6.0 were used for tigecycline [20].

Clonal diversity was performed by pulsed-field gel electrophoresis (PFGE), as previously described [21], with some modifications. Chromosomal DNA from the isolates was digested with 10 U of *XbaI* (Takara Bio Inc., Shiga, Japan) and electrophoresis was performed on the CHEF-DR III system (Bio-Rad Laboratories, Hercules, CA, USA) at 6 V/cm, with switch time of 0.5 s to 30 s for 23 h at 14 °C. Pulsed-field gel electrophoresis patterns were analyzed visually, and classified as suggested by Tenover *et al*. [22].

Plasmid profiles were screened by the method described by Kieser [23], and plasmid sizes were determined by comparison with *E*. *coli* NCTC 50912. The horizontal transfer of carbapenem resistance was performed by bacterial conjugation with *E*. *coli* J53 as the recipient strain [17, 24]. Transconjugants were selected on Luria-Bertani (LB) agar supplemented with sodium azide (100 μg/mL) plus cefoxitin (30 μg/mL). The confirmation of the transfer of carbapenem resistance was made by antimicrobial susceptibility and PCR for transconjugants strains.

The incompatibility groups were detected by PCR-based replicon typing in the transconjugant [25, 26]. Multilocus Sequence Typing (MLST) was carried out in selected NDM-1-producing *K*. *pneumoniae* isolates [27] and analyzed using the *K*. *pneumoniae* MLST website (http://bigsdb.pasteur.fr/klebsiella/klebsiella.html).

#### Ethics

The local ethics committee (Comité de Ética y Comité de Investigación, Facultad de Medicina y Hospital “Dr. José Eleuterio González,” Universidad Autónoma de Nuevo León, Mexico) and the infection prevention and control committee approved the study procedures. Informed consent was obtained from the participants before the collection of samples. Verbal informed consent was obtained from the health care personnel before the collection of samples, participation was voluntary, and only staff that was willing to participate after an explanatory meeting with the infection control team were included. The patients or caregivers (for those not able to consent) provided written informed consent for this study.

## Results

### Follow-up and control of the outbreak

During the outbreak a total of 240 cultures were taken and processed, and of those, 24 (9.8%) were from hospital surfaces and devices, 65 (26.5%) were from healthcare workers, and 151 (61.6%) were from patients (67 patients in total). All of the ICU patients were sampled (63/67), as well as the patients that shared a room or were in contiguous rooms from the source patient in the neurology ward (4/67). All samples from the environment and healthcare workers were negative.

After the three patients (P1, P2, and P3) were discharged from the ICU, there were no more positive results for CRE after 3 weeks. The rectal swabbing was stopped, and the unit returned to the standard protocols for infection control. Eight months after the alarm was ended, no more CRE have been detected through routine surveillance.

### Microbial growth and additional assays

In all rectal swabs, microbial growth was observed with a predominance of rectal and cutaneous microbiota, including *Enterococcus* sp., Coagulase-negative Staphylococci, *Corynebacterium* sp., *Chryseobacterium* sp. *E*. *coli*, *K*. *pneumoniae*, *Citrobacter* sp., *Enterobacter* sp., *Proteus* sp., *Acinetobacter* sp., and *Pseudomonas* sp. At least five different colonies of each patient were identified using MALDI-TOF.

During the surveillance, a total of 10 carbapenem-resistant and carbapenemase-producing *K*. *pneumoniae* isolates were collected (eight from P1 and one from P2 and P3 each). All 10 isolates were positive for the *bla*_NDM-1_ gene.

In addition, they were positive for extended-spectrum β-lactamases (ESBL) *bla*_TEM-type_, *bla*_SHV-type_, and *bla*_CTX-type_ and were negative for *bla*_CMY-2._

**Fig 1:**
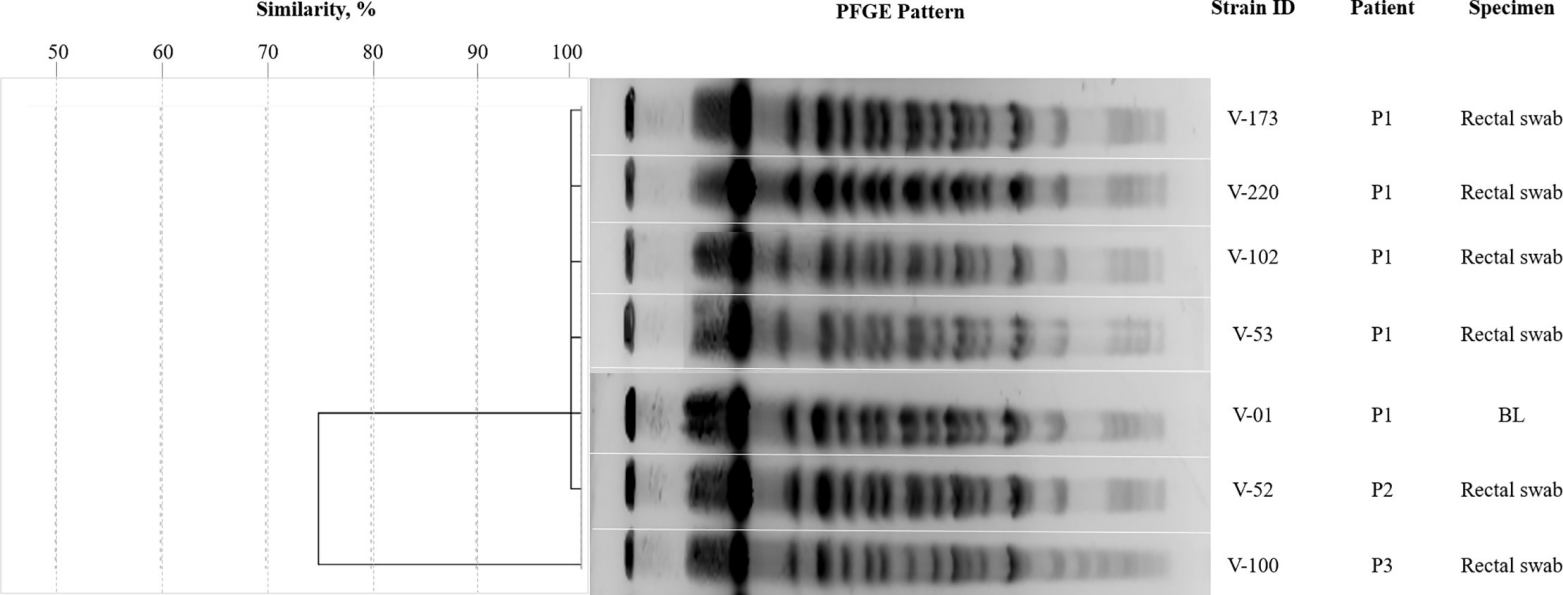
Dendogram and PFGE patterns of NDM-1-producing *K*. *pneumoniae* isolates. P1 represents patient 1; P2 represents patient 2; P3 represents patient 3; and BL represents bronchoalveolar lavage.

Pulsed-field gel electrophoresis demonstrated that P1 and P2 isolates had 100% similarity and P3 isolate had 85% similarity (Fig 1). All 10 isolates displayed the same susceptibility pattern of being resistant to amikacin, meropenem (> 32 μg/mL), cefoxitin, ceftazidime, cefepime, ampicillin (> 16 μg/mL), gentamicin, imipenem (> 8 μg/mL), levofloxacin, ceftriaxone (> 4 μg/mL), ciprofloxacin (> 2 μg/mL), ertapenem (> 1 μg/mL), ampicillin/sulbactam (> 16/8 μg/mL), and piperacillin/tazobactam (> 64/4 μg/mL), with susceptibility only for tigecycline (2 μg/mL) and colistin (2 μg/mL).

**Fig 2:**
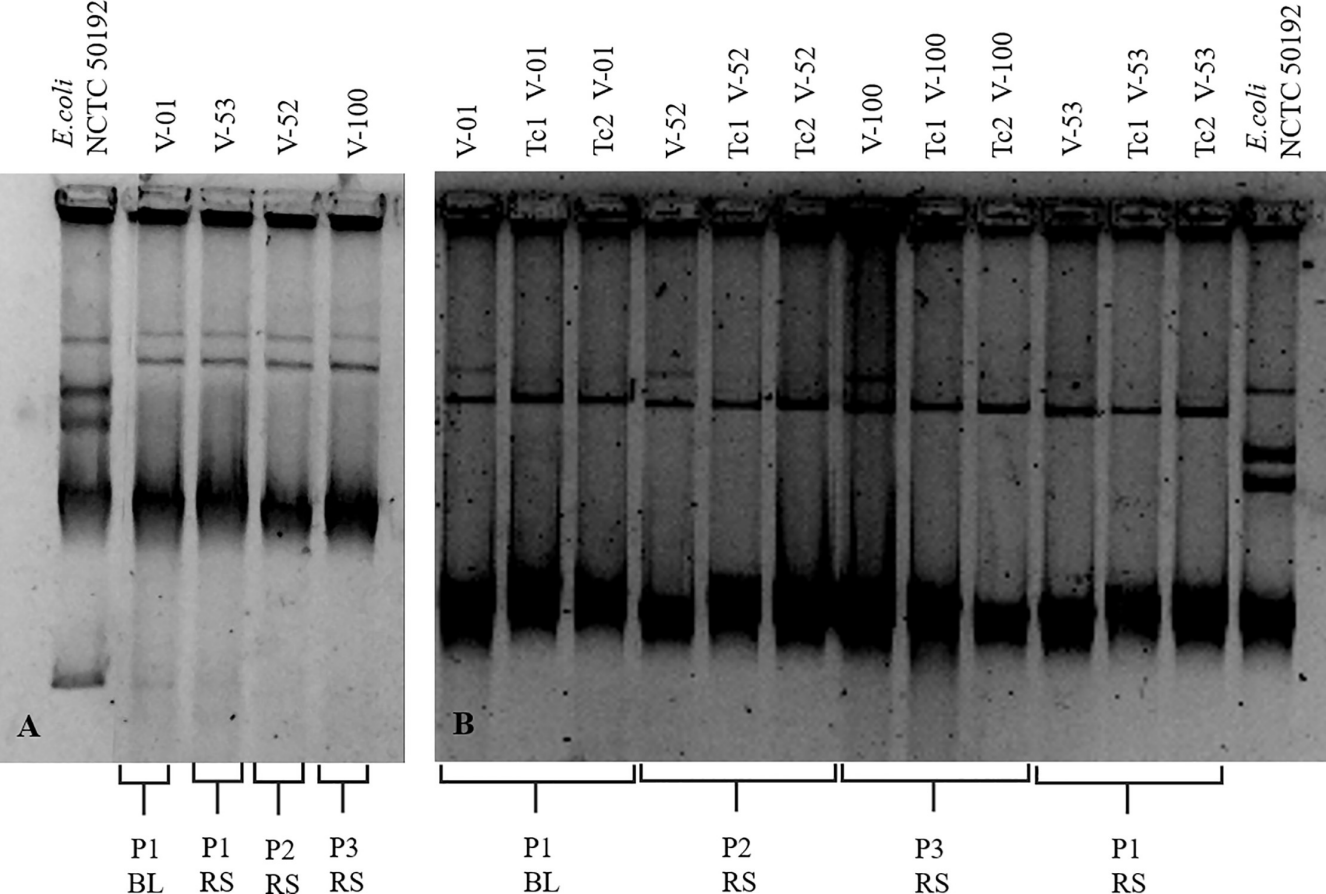
Plasmid profiles of the NDM-1-carrying *K*. *pneumoniae* and transconjugants isolates. (A): Plasmid profile of clinical isolates; (B): Plasmid profile of clinical and transconjugants isolates; Tc represents transconjugants; P represents patient; BL represents bronchoalveolar lavage, and RS represents rectal swab.

The NDM-1-producing *K*. *pneumoniae* isolates revealed the same plasmid patterns with 130-kb and 150-kb plasmids. The mating experiments were analyzed and were successful in all isolates (Fig 2); the *bla*_NDM-1_ gene was transferred onto a 130-kb conjugative plasmid belonging to the incompatibility groups IncY, IncFIIs, and IncFIIY. The MLST analysis of *K*. *pneumoniae* identified the ST307 in four selected NDM-1-producing *K*. *pneumoniae* isolates (two from P1 and one from P2 and P3 each).

### Comparison of methodologies used

When comparing the direct PCR from the broth as a rapid method for detection of carbapenemase genes with detection from colonies, we included 167 results in which we had both results of direct PCR and results from culture. Among them, we had 8 true positives, 167 true negatives, 1 false negative and 0 false positive. Direct PCR had a sensitivity of 88.9% (51.75–99.72; 95% CI), specificity of 100% (97.82–100.00; 95% CI), PPV of 100%, and NPV 99.40% (96.34 to 99.91; 95% CI) and accuracy 99.43% (96.88 to 99.99; 95%CI). The *kappa* between the direct PCR from the broth and the CarbaNP test was 0.938.

## Discussion

In this study, the successful containment of a hospital outbreak caused by NDM-producing *K*. *pneumoniae* ST307 is described, and, as is common in the control of outbreaks, the researchers were unaware that they were dealing with the *K*. *pneumoniae* ST307, which has been described as a high-risk clone [12, 28–30], both in KPC-producing and NDM-producing *K*. *pneumoniae* isolates [12]. The first detection of NDM-1-producing *K*. *pneumoniae* ST307 was in 2008, and it has emerged worldwide, with outbreaks in the United States in 2013 [30], China, Tunisia [31], Italy, Korea, Pakistan, Morocco [28], and Mexico [32].

In Mexico, an outbreak in 2017, including 46 carbapenem-resistant *K*. *pneumoniae* isolates, was reported in a third-level hospital in Jalisco. In this outbreak, the predominant clone belonged to ST392, but ST307, ST309, ST846, and ST2399 were also detected [32]. Likewise, the *K*. *pneumoniae* ST307 clone has been associated with the production of ESBL CTX-M-15 and KPC-2 [12, 28–30]. The clones detected in this report harbor the ESBL CTX-type and were negative for KPC-2.

Human error played an initial key role; the hospital´s protocol requires that every patient transferred from another institution be cultured from potential sites of infection or colonization during the first 24 hrs., the infection control personnel routinely orders this, however in the initial patient cultures were sent after 3 days of hospital stay and the interpretation of the results was initially misclassified, this delay might have been linked to the spread of *K*. *pneumoniae bla*_NDM_ to the other patients. Retraining of personnel and an enhancement in the protocol including rectal swabbing for detection of carbapenem-resistant enterobacteria were performed during the outbreak and are constantly reinforced. Also, once the outbreak started, vital actors, such as hospital administrators, nursing staff, heads of departments, caregivers, microbiologists, and infection control specialists working in conjunction, were necessary to control it.

During the outbreak, enhanced infection control procedures were put in place including that all patients in the ICU, the neurology and patients that had been in the same period and discharged from the ICU were put under contact precautions. It should be considered that other uncontrolled factors or chance may have played a role in stopping onwards transmission.

The worldwide dissemination of high-risk carbapenemase-producing *K*. *pneumoniae* clones has become a significant threat to healthcare facilities. The present work could be part of an epidemiology alert in Mexico of the possible dissemination of the clone NDM-producing *K*. *pneumoniae* ST307, because of this second report of this clone in this country.

Carbapenem resistance and carbapenemase production have been detected in this hospital in *Acinetobacter baumannii* (*bla*_OXA-24_ and *bla*_OXA-58_); [33] and *Pseudomonas aeruginosa* (*bla*_IMP_; data not published), with *P*. *rettgeri* (*bla*_NDM-1_) being the only CRE identified in this hospital until this outbreak [7].

One of the limitations of this study is that we do not have the complete genome sequence of the *K*. *pneumoniae* NDM strains recovered thus, which would have allowed us to infer inter-patient transmission with more confidence. Thus, it may be appropriate to consider it as a possible intrahospital transmission rather than an outbreak. However, some epidemiological data support that was actually an outbreak *e*.*g*. a) Patients 2 and 3 had no history of hospitalization and had not been in contact with other healthcare centers, b) Patients 1, 2 and 3 were in the ICU sharing medical personnel, c) the hospital has no history of *K pneumoniae* NDM infections before this event, d) in less than two weeks, 3 epidemiologically related cases were detected and the strains have the same ST, plasmid profile, antibiotic resistance pattern and were PFGE related.

PFGE/MLST results and epidemiological data suggest the presence of an outbreak, however, the small-scale chain of transmission reported here may be the result of the spontaneous resolution of this event.

In this study, we compared the results of direct PCR from the broth as a rapid method for detection of carbapenemase genes with detection from colonies in culture, and we detected that direct PCR had a sensitivity of 88.9% and the *kappa* value between the direct PCR from the broth and the CarbaNP test was 0.938. The results of sensitivity and accuracies should be interpreted cautiously, because of the low number of positive specimens detected and that only one carbapenemase type was detected.

In this outbreak, the results of PCR for NDM gene directly from mixed broth were obtained approximately 15 h after swabbing the patient. When a positive result was observed, the infection control unit´s staff was informed in no more than 10 min, and they immediately did the epidemiological analysis (because we were unaware if P2 or P3 were the original carriers) and additional measures were implemented when needed.

No dissemination of the clone in the hospital environment was detected after 3 weeks (no positive cultures), and, importantly, no time was allowed for the colonization of the healthcare workers.

In conclusion, it was possible to contain a hospital outbreak caused by NDM-producing *K*. *pneumoniae* ST307 through active epidemiological and microbiological surveillance. With the methodology used, the detection of the NDM gene in fecal samples was obtained in approximately 15 hours after obtaining the fecal sample.

## Supporting information

S1 Table Primers for amplification and sequencing of carbapenemase-encoding genes. (DOCX)
