# Supplementary material for: The successful containment of a hospital outbreak caused by NDM-1-producing Klebsiella pneumoniae ST307 using active surveillance
Source: PLoS One. 2019 Feb 13;14(2):e0209609. doi: 10.1371/journal.pone.0209609 (PMC6373926; doi:10.1371/journal.pone.0209609)
Supplement: S1 Table — (DOCX) [file pone.0209609.s001.docx]

| Primer name | Primer sequence (5´→3´) | Annealing temp (°C) | Product (pb) | Reference | |
| --- | --- | --- | --- | --- | --- |
| KPC F | GCAGCGGCAGCAGTTTGTTGATT |  |  |  |  |
| KPC R | GTAGACGGCCAACACAATAGGTGC | 60 | 184 | 16 |  |
| IMP-F | GGAATAGAGTGGCTTAAYTCTC |  |  |  |  |
| IMP-R | CCAAACYACTASGTTATCT | 52 | 188 | 17 |  |
| NDM-1F | GGAAACTGGCGACCAACG |  |  |  |  |
| NDM-1 R | ATGCGGGCCGTATGAGTGA | 60 | 678 | 18 |  |
| pre-NDM-for* | CACCTCATGTTTGAATTCGCC |  |  |  |  |
| pre-NDM-rev* | CTCTGTCACATCGAAATCGC | 56 | 984 | 21 |  |
| VIM-F | ATGGTGTTTGGTCGCATATC |  |  |  |  |
| VIM-R | TGGGCCATTCAGCCAGATC | 60 | 510 | 19 |  |
| OXA-48 F | TTCGGCCACGGAGCAAATCAG |  |  |  |  |
| OXA-48 R | GATGTGGGCATATCCATATTCATCGCA | 62 | 240 | 16 |  |

**S1 Table.** Primers for amplification and sequencing of carbapenemase-encoding genes

*Primers used for sequencing of NDM gene
